# Predicting the Risk of Hearing Impairment Following the Cervical Spine Diseases by Measuring the Cervical Range of Movements: A Pilot Study

**DOI:** 10.18869/nirp.bcn.8.5.413

**Published:** 2017

**Authors:** Behnoosh Vasaghi-Gharamaleki, Zahra Naser

**Affiliations:** 1. Department of Basic Sciences, School of Rehabilitation Sciences, Iran University of Medical Sciences, Tehran, Iran.; 2. Rehabilitation Research Center, Iran University of Medical Sciences, Tehran, Iran.; 3. Department of Audiology, School of Rehabilitation Sciences, Iran University of Medical Sciences, Tehran, Iran.

**Keywords:** Cervical neck injuries, Hearing loss, Range of motion, Cervical movement

## Abstract

**Introduction:**

Cervical spine abnormalities can affect the ear vessels and or nerves with different mechanisms. Ear dysfunctions following cervical spine injuries can be manifested as hearing loss, vertigo, or tinnitus. Usually, cervical spine injuries can cause pain and Range of Motion (ROM) limitation. The major objective of this study was to determine which cervical ROM limitation was accompanied with higher level of hearing loss.

**Methods:**

In this cross-sectional study, 42 volunteers (20 women and 22 men) with cervical spine injury and pain participated after giving their informed consent. Audiometry, tympanometry, and pure-tone threshold of individuals were taken in frequencies from 250 to 8000 Hz in all cases. The ROM limitation in flexion, extension and rotation was recorded.

**Results:**

About 53% of participants had bilateral hearing loss. In 40.48% (n=17) of cases, rotation to the left was limited. Flexion and extension motion were restricted on 23.8% (n=10) and 30.95% (n=13) of the participants, respectively. There was no statistically significant relationship between sex and hearing loss but a significant correlation was observed between hearing loss and ROM limitation of rotation to the left in men.

**Conclusion:**

According to the present study, the likelihood of hearing loss was high in patients with cervical left rotation limitation, and that the incidence of hearing loss following the cervical spine injuries was more in men. It seems that left Rotation limitation can be used as a predictor to diagnosis of hearing impairment following the cervical spine injuries (especially in men).

## 1. Introduction

The neck consists of seven vertebrae and is most prone to spinal injuries after the lumbar area. The cervical spine is divided into the upper and lower parts. The upper neck consists of the occiput and the first and second vertebrae, and the lower neck consists of the third to seventh vertebrae (Motsitsi & Bomela, 2009). Previous studies have shown that any abnormality of the cervical spine (especially upper cervical spine) can affect the arteries or nerves related to the ear and disturb their function through different mechanisms. For instance, 60% of the patients with cervical rheumatoid arthritis suffer from hearing disorders (Reiter, Konkle, Myers, Schimmer, & Sugar, 1980). The hearing impairment following the cervical spine diseases (such as osteoarthritis, rheumatoid arthritis, disk herniation at cervical upper segments, whiplash, etc.) may appear as hearing loss, vertigo, and tinnitus (Galm, Rittmeister, & Schmitt., 1998; Ozturk et al., 2004; Rawool & Harrington, 2007; Murdin, Patel, Walmsley, & Yeoh, 2007). Usually, the treatment or rehabilitation of the condition reduces the hearing loss or obviates vertigo and tinnitus. The less the time of cervical spine involvement, the faster and more favorable the symptoms of the disease would be resolved (Kessinger & Boneva, 2000).

Cervical spine diseases usually cause pain and patients usually avoid moving and suffer from limitation of movement. Meanwhile, the inflammatory nature of many diseases may cause adhesion in the soft tissue and decrease the range of physiological movements of the cervical spine. There are four general categories of physiological movements for the cervical spine as follows: flexion, extension, bending, and rotation (Voss, Page, & Benger, 2012).

All studies on the incidence of hearing loss in cervical spine injuries and diseases only examined the presence of an injury and its effects on auditory system (such as, hearing loss and vertigo) (Galm et al.,1998; Ozturk et al., 2004; Rawool & Harrington, 2007; Murdin et al., 2007). The major objective of this pilot study was to determine which cervical Range Of Movement (ROM) limitation was accompanied with a higher level of hearing loss. In other words, can we provide a test for identifying patients at risk of developing hearing loss after cervical spine diseases?

## 2. Methods

Study Participants were recruited from Rheumatology and Orthopedic outpatient clinics at Hazrat-e Rasool Hospital. Finally, this cross-sectional study was performed on 42 patients who suffered from cervical injury and neck pain, and volunteered to participate in this study with informed consent. The present study was approved by the Ethics Committee of Iran University of Medical Sciences. The patients underwent audiometry tests after the doctor diagnosed and confirmed their cervical spine injuries (including osteoarthritis, disk herniation at cervical upper segments, spondylolysis, intervertebral disk protrusion, or a combination of these conditions). A questionnaire including general information on age, sex, and duration of neck pain was completed for all patients.

The normal hearing standard in all studied patients was the pure-tone hearing threshold equal to or higher than 25 dB HL at octave frequencies of 250 to 8000 Hz in both ears. The normal results of tympanometry were chosen as follows: Canal size of 0.9–2.0 cm^3^; Static compliance of 0.2–1.5 m℧; and sound pressure of ±50 daPa (Katz, Medwetsky, Burkard, & Hood, 2009). The Word Discrimination Score (WDS) was chosen to be in the normal range. The patients underwent tympanography and pure-tone audiometry threshold test at frequencies of 250–8000 Hz. During all tests, patients’ head was positioned in the midline. The presence of tinnitus, vertigo, and dizziness was examined and recorded through an interview.

The ROM of cervical spine was measured according to routine physical examination (Voss et al., 2012). The examiner performed each movement for the patient and then asked the patient to do so as described below to measure the range of flexion (Flex), extension (Ext), and rotation (Rot) of cervical spine. To do the Flex movement, patients were asked to look forward and bend down their head until the chin met the chest. To measure the range of Ext movement, the patients were asked to sit down, look forward, and draw back their head as the chin was abducted from the chest, the back of the head met the back, and the patients could not draw back their head anymore. To measure the range of Rot movement, the patients were asked to sit down, look forward, and rotate their head once to the right and once to the left from the midline. In each rotation to either side, the chin should be positioned parallel to the shoulder of that side. Failure to complete any of the above movement was recorded as the limitation for that movement. In all movements, the patients were asked to move only the neck and sit with their back touching the back of the chair (Voss et al., 2012).

The results are presented as mean and standard deviation. The data were analyzed using SPSS (version 18). The statistical methods comprised ANOVA, independent t test and the Chi-square test, at significance level of α≤0.05.

## 3. Results

This study was performed on 22 men with the mean(SD) age of 41.1(13.86) years and 20 women with the mean(SD) age of 45.21(11.75) years. Almost 50% of the participants were over 45 years old. Tinnitus, vertigo, and dizziness were respectively observed in 50%, 48.5%, and 48% of the participants. About 53% of cases had bilateral hearing loss. The limitation of Flex, Ext and, left Rot movements was respectively observed in 23.8% (n=10), 30.95% (n=13) and 40.48% (n=17) of patients. The examination of the cervical movements did not show any limitation at right Rot.

The Chi-square test did not show a significant difference between the two sexes in terms of the presence or absence of limitation in cervical Flex, Ext, and Rot movements. The limitation in cervical Flex, Ext, and Rot movements did not show a statistically significant relationship with the reduced static compliance of both ears. There was no statistically significant relationship between cervical movement limitation and vertigo/dizziness. However, there was a statistically significant relationship between limitation of left Rot movement and hearing loss only in men (Table 1).

**Table 1.** The mean and standard deviation measurements of pure-tone audiometry in physiological ROM of cervical spine movements.

**Test Frequency (Hz)**		**Extension**						**Flexion**						**Rotation to the Left**					

		**Female**		**P**	**Male**		**P**	**Female**		**P**	**Male**		**P**	**Female**		**P**	**Male**		**P**
					
		**+(N=7)**	**−(N=13)**		**+(N=7)**	**−(N=15)**		**+(N=5)**	**−(N=15)**		**+(N=6)**	**−(N=16)**		**+(N=6)**	**−(N=13)**		**+(N=9)**	**−(N=12)**	
Right ear	250	21.43 (±13.45)	21.92 (±14.07)	0.940	25.71 (±22.44)	18.00 (±10.82)	0.415	21.00 (±13.42)	22.00 (±13.99)	0.890	28.33 (±23.38)	17.50 (±10.65)	0.318	21.67 (±12.52)	22.69 (±14.52)	0.883	28.33 (±20.62)	15.83 (±6.69)	0.113
	500	23.57 (±13.76)	24.61 (±15.87)	0.885	25.00 (±22.91)	16.67 (±10.47)	0.388	25.00 (±10.00)	24.00 (±16.39)	0.900	28.33 (±23.38)	15.93 (±10.36)	0.259	23.33 (±11.25)	25.77 (±16.56)	0.749	28.33 (±20.77)	13.33 (±5.37)	0.064
	1000	27.86 (±14.39)	21.54 (±16.38)	0.403	25.00 (±23.09)	19.67 (±12.17)	0.582	23.00 (±8.37)	24.00 (±17.65)	0.905	30.00 (±22.14)	18.13 (±12.50)	0.259	24.17 (±7.36)	24.23 (±18.91)	0.992	31.11 (±19.97)	15.00 (±8.26)	0.046
	2000	27.14 (±14.39)	22.69 (±16.41)	0.555	27.14 (±28.41)	20.33 (±14.33)	0.562	22.00 (±9.10)	25.00 (±17.32)	0.718	30.83 (±28.88)	19.38 (±14.59)	0.389	25.83 (±11.14)	24.23 (±17.89)	0.844	35.00 (±23.45)	13.75 (±10.25)	0.029
	4000	34.29 (±14.56)	25.38 (±18.08)	0.278	36.43 (±21.55)	30.67 (±17.82)	0.515	31.00 (±10.84)	27.67 (±18.98)	0.717	36.67 (±24.01)	30.94 (±17.05)	0.609	33.33 (±11.25)	26.92 (±19.74)	0.383	43.33 (±23.32)	25.42 (±10.54)	0.056
	8000	37.14 (±24.30)	28.08 (±18.66)	0.363	34.29 (±21.88)	38.00 (±22.26)	0.718	34.00 (±24.08)	30.33 (±20.22)	0.741	41.67 (±23.17)	35.00 (±21.60)	0.556	40.00 (±22.36)	28.08 (±20.16)	0.262	47.22 (±26.23)	28.75 (±15.39)	0.084
Left ear	250	25.71 (±14.56)	21.92 (±17.14)	0.626	23.57 (±17.25)	18.33 (±13.32)	0.443	23.00 (±11.51)	23.33 (±17.59)	0.962	26.67 (±16.93)	17.50 (±13.17)	0.193	24.17 (±13.19)	23.85 (±17.81)	0.969	28.89 (±18.67)	14.17 (±5.97)	0.048
	500	26.43 (±13.45)	21.92 (±17.26)	0.558	24.29 (±23.53)	16.33 (±13.95)	0.330	21.00 (±9.62)	24.33 (±17.61)	0.695	28.33 (±23.38)	15.31 (±13.84)	0.120	24.17 (±10.68)	24.23 (±18.24)	0.994	31.11 (±21.47)	10.83 (±5.57)	0.022
	1000	30.00 (±12.91)	21.54 (±18.53)	0.299	23.57 (±25.45)	17.00 (±12.80)	0.538	23.00 (±9.08)	25.00 (±19.09)	0.758	28.33 (±25.43)	15.63 (±12.67)	0.287	28.33 (±10.80)	24.23 (±19.13)	0.633	32.78 (±19.22)	10.00 (±7.07)	0.007
	2000	30.71 (±14.84)	22.31 (±17.87)	0.303	25.71 (±25.07)	20.00 (±17.11)	0.536	22.00 (±9.75)	26.33 (±18.94)	0.519	27.50 (±26.97)	19.69 (±16.58)	0.417	30.83 (±10.21)	23.85 (±19.38)	0.422	35.56 (±22.42)	12.08 (±10.10)	0.015
	4000	35.71 (±13.67)	24.62 (±18.42)	0.180	37.14 (±23.78)	33.33 (±24.69)	0.737	32.00 (±11.51)	27.33 (±19.17)	0.526	36.67 (±26.77)	33.75 (±23.63)	0.806	36.67 (±10.80)	26.15 (±19.06)	0.228	45.00 (±25.00)	28.33 (±21.67)	0.119
	8000	39.29 (±24.90)	26.54 (±19.51)	0.221	35.00 (±25.66)	38.00 (±28.77)	0.817	40.00 (±28.06)	28.00 (±19.53)	0.299	43.33 (±27.33)	34.69 (±27.72)	0.521	43.33 (±24.01)	26.54 (±19.83)	0.126	49.44 (±29.42)	26.25 (±22.47)	0.054

+: With ROM limitation; −: Without ROM limitation; Significant results are bold; P<0. 05; ROM: Range of motion.

No statistically significant correlation was found between sex and hearing loss when the studied frequencies were divided into two categories, speech frequencies (500–2000 Hz) and high frequencies (4000–8000 Hz). The statistical analysis of the above frequencies and presence/absence of movement limitation showed a significant difference in left Rot limitation (except the range 500–2000 Hz) (P<0.05). Most patients in this study were using sedatives and rehabilitative treatment to relieve the cervical pain; however, the amount and type of drugs were not recorded precisely.

## 4. Discussion

The present study showed that among patients with cervical spinal diseases, those with left Rot limitation had higher odds of hearing loss than those who did not, and that the prevalence of hearing loss following spinal diseases was more among men than among women. It seems that left Rot limitation can be used as a predictor of hearing impairment following the cervical spine diseases (especially in men). Sensorineural hearing loss can be caused by the inflammation of the blood vessels and nerves, the involvement of the middle ear bones, ligament injuries, or the effects of ototoxic drugs for relieving pain (salicylates and NSAIDs) in patients with cervical injuries (Rawool & Harrington, 2007; Seçkin, et al. 2000).

Öztürk et al. study showed bilateral hearing loss in patients with cervical disorders (Ozturk et al., 2004). The present study also showed a bilateral hearing loss in 53% of the patients and, in conformity to Yamasoba et al. study, determined the level of hearing loss as mid- to high-tone loss (Kumral, Kisabay, & Atac, 2006). Most abnormalities and disorders of cervical spine can cause disorders in the alignment of the vertebrae, especially the upper vertebrae, and damage or put pressure on vertebral and basilar arteries (Ozturk et al., 2004). The vertebrobasilar artery ischemia, especially the occlusion of anterior inferior cerebellar artery or one of its branches, regardless of the age, can cause bilateral hearing loss (Amarenco & Hauw, 1990; Yamasoba, Kikuchi, & Higo, 2001; Kumral, et al., 2006).

The movement limitation is one of the earliest symptoms of cervical spine injuries. Although various studies revealed the effect of cervical injuries on hearing threshold (Galm et al., 1998; Ozturk et al., 2004; Rawool & Harrington, 2007; Murdin et al., 2007), in this study, we tried to find which movement limitation following the cervical spine injuries was associated with higher hearing loss and could use it as a predictor of auditory system injury. Studies have shown that the gradual degradation of the intervertebral disk and articular cartilage was the major cause of the chronic neck pain in the elderly, often caused the dysfunction of spinal nerves, and largely reduced the range of cervical movement, especially in cervical Rot movement (Rao, 2003; Wu et al., 2013).

Movement limitations of cervical spine mostly seen in people over 35 years and for different reasons (Hasler et al., 2012). As the mean age of almost 50% of the study patients was over 45 years, the incidence of cervical injuries might be associated with their age, too. On the other hand, the Rot movement mostly occurs at upper neck and its limitation may indicate that the nerves and blood vessels are at risk of damage. Studies have shown that improving cervical Rot movement relieved the pain in patients with neck pain (Cassidy, Quon, LaFrance, & Yong-Hing, 1992; Wood, Colloca, & Matthews, 2001). Because pain suggests a damage, causes of pain also can limit Rot and affect hearing loss.

The results showed no significant differences between mean hearing thresholds of men and women with limitations in Flex and Ext movements and those without limitations. However, there was a significant difference between mean hearing threshold of men with limitations in left Rot movements and those without limitations (Table 1). As mentioned before, it appears that the factors that cause limitation of motion in the neck rotation following the cervical spine diseases can also cause hearing impairment. Unfortunately, this study did not check subjects’ left- or right-handedness. However, according to the prevalence of right-handedness, the limitation in left Rot might have been due to being right-handed. Examining this factor appears necessary in order to achieve a better understanding of the effects of being right-handed on the hearing impairment following the cervical spine diseases. Despite hearing impairment in almost all the studied frequencies, only the speech frequencies (frequency of 500 to 2000, especially in the left ear) had shown significant differences. This can indicate the presence of problems in verbal communication of these people. Therefore, it is recommended that this issue be addressed in future research.

It has been shown that women had more favorable hearing threshold than men, and their right ears hearing responses were better than those of the left ears (McFadden, 2009; McFadden, Pasanen, Leshikar, Hsieh, & Maloney, 2012). The present study showed a higher level of hearing loss in men, as the level of hearing loss in the left ear was higher than that in the right ear, and in case of any limitation at cervical movements, the level of hearing loss in the left ear was higher than that in the right ear (Table 1). These results might confirm the influence of sex on the incidence of hearing loss following movement limitation due to the cervical injuries. It has to be borne in mind that these results might be clinically less valuable and more studies are needed for more valid results.

In this study, vertigo and dizziness were observed in many patients. Vertigo and hearing loss are mostly caused by dysfunction of the upper cervical vertebrae, and this is a common problem in the fields of ear, nose, and throat, and neurological diseases (Galm et al., 1998; Haldeman & Dagenais, 2001). It seems that the dysfunction at segments lower than C3 (lower neck) cannot cause hearing loss or vertigo. Most of the cervical Rot is also occur in the upper neck and the Rot limitation may cause vertigo and dizziness associated with hearing loss (Galm et al., 1998; Haldeman & Dagenais, 2001) as seen in this study.

One of the limitations of this pilot study relates to ROM limitation which was recorded only as presence or absence. Therefore, future studies are recommended to check degree of ROM and how it affects the level of hearing loss. In addition, further studies should be performed to clarify whether hearing loss occurs after movement limitations in patients with muscular diseases (not bone diseases) of the neck (such as trigger point).

Based on the results, in the event of injury to the cervical spine following degenerative diseases, the left Rot limitation could predict the occurrence of hearing impairment (especially in men) among the various mobility restrictions that arise in the neck.
